# Newcastle disease virus genotype VII gene expression in experimentally infected birds

**DOI:** 10.1038/s41598-022-09257-y

**Published:** 2022-03-28

**Authors:** Phuong Thi Kim Doan, Wai Yee Low, Yan Ren, Rick Tearle, Farhid Hemmatzadeh

**Affiliations:** 1 School of Animal and Veterinary Sciences, The University of Adelaide, Adelaide, Australia; 2 Faculty of Animal and Veterinary Sciences, Tay Nguyen University, Dak Lak, Vietnam; 3 Davies Research Centre, School of Animal and Veterinary Sciences, The University of Adelaide, Adelaide, Australia

**Keywords:** High-throughput screening, Pathogens, Virology, Transcription, Transcriptomics, Gene expression, Gene regulation, Genome, Genomics, Sequencing, Sequencing, Bioinformatics, Gene expression analysis, Genetic techniques, Genomic analysis, High-throughput screening

## Abstract

Newcastle disease virus genotype VII (NDV-GVII) is a highly contagious pathogen responsible for pandemics that have caused devastating economic losses in the poultry industry. Several features in the transcription of NDV mRNA, including differentially expressed genes across the viral genome, are shared with that for other single, non-segmented, negative-strand viruses. Previous studies measuring viral gene expression using northern blotting indicated that the NDV transcription produced non-equimolar levels of viral mRNAs. However, deep high-throughput sequencing of virus-infected tissues can provide a better insight into the patterns of viral transcription. In this report, the transcription pattern of virulent NDV-GVII was analysed using RNA-seq and qRT-PCR. This study revealed the transcriptional profiling of these highly pathogenic NDV-GVII genes: NP:P:M:F:HN:L, in which there was a slight attenuation at the NP:P and HN:L gene boundaries. Our result also provides a fully comprehensive qPCR protocol for measuring viral transcript abundance that may be more convenient for laboratories where accessing RNA-seq is not feasible.

## Introduction

Newcastle disease virus is a member of the *Avian orthoavulavirus 1* species in the genus *Orthoavulavirus* subfamily A*vulavirinae*, and family *Paramyxoviridae*^1^*.* NDV strains possess a single-stranded, non-segmented and negative-sense RNA genome that is approximately 15,000 nucleotides in length. The genome contains six major genes encoding six proteins: nucleocapsid protein (NP); phosphoprotein (P); matrix protein (M), fusion protein (F); hemagglutinin-neuraminidase protein (HN) and large polymerase protein (L) in the order 3′-NP-P-M-F-HN-L-5′. Additionally, two non-structural proteins V and W are derived from P^2,3^ through RNA editing. HN and F encode two surface glycoproteins and are responsible for virus entry and budding, respectively. M protein is required for the integrity of the virus particles and is located on the inner surface of the envelope, whereas NP, P and L proteins constitute a viral RNA-dependent RNA polymerase (RdRP) complex that has a crucial role in RNA transcription and synthesis^2,4^. In addition, V protein modulates the viral RNA replication through inhibition of host IFN signalling^5^, whereas the information of the W protein function is limited. The W protein of Nipah virus plays an important role in viral pathogenesis and supports the virus to evade the host IFN-activity^6^.

Like other paramyxoviruses, NDV uses its negative-sense genome as the template for the viral RNA transcription to generate six separate viral transcripts^4,7^. The 3′ and 5′ end of the genome contains extra-cistronic regions of 55nt and 114nt which make up the 3′ leader and 5′ trailer region, respectively. Transcription occurs when RdRP recognises and attaches to the leader promoter and scans along the genome until it reaches the first gene start (GS) site, where NP gene transcription is initiated. It appears that capping and methylation are carried out by RdRP via a signal from the GS site and polyadenylation occurs after the gene end (GE) site^4,8^. The 5′ capped, methylated and 3′ polyadenylated viral transcript is then released by the RdRP. After terminating the transcription of an upstream mRNAs, RdRP either disengages from the genome at a GE site and reinitiates at the leader promotor to continue transcribing or traverses the intergenic region and reinitiates at a GS site of the next gene to transcribe. The viral mRNAs of negative strand RNA viruses are most likely synthesized by a sequential and discontinuous mechanism involving polymerase stop-start signals, which terminate at the end of each preceding gene and restart at the beginning of the next gene^4,9–11^. Because not all RdRP reinitiate, the mRNA is more abundant for genes nearer the 3′ end compared with those nearer the 5′ end of the viral genome^12^, a phenomenon known as a transcriptional gradient^2^.

However, this process is not always efficient and RdRP occasionally fails to terminate the transcription at the gene-end site, which leads to the transcription of mRNA across the intergenic region and downstream gene(s), producing different loads of mRNA^13,14^. The viral gene expression of several paramyxoviruses such as Measles, Hendra, Sendai, Mumps virus and Parainfluenza virus type 2, 3, 5 have been studied^8,13–15^. Transcriptional gradients of viral mRNAs and steep attenuation of transcription occur at M-F and G-L junctions of Hendra virus, at M-F and HN-L junctions of Sendai virus and at NP-P and HN-L junctions of Measles virus. Furthermore, the transcription attenuation varies between different strains of vesicular stomatitis virus (VSV), another non-segmented, negative-strand RNA virus^16^. An isolate with a small plaque phenotype was found to have a steeper transcription gradient and to generate fewer mRNA transcripts than the wild-type virus^16^. Similarly, the transcription patterns of the respiratory syncytial and Ebola viruses is genotype dependent, but was in an non-transcriptional gradient pattern^17,18^, suggesting gene expression mechanisms of pathogenic viruses are variable.

Non-equimolar amounts of NDV polypeptides are produced both in vitro and in vivo^11^. However, the quantification of viral transcripts using Northern blotting appears to be relatively less intensive , so high throughput sequencing (HTS) has been used to measure the viral mRNAs after viral infection^8,19^. Using counts per million of viral transcripts as the measure, there is a gradual decrease in amounts from the 3’ to 5’ end of the genome in the trachea epithelial cells^20^. In contrast, there were higher viral mRNA amounts of F and HN genes found in the Harderian gland of challenged birds. In addition, the quantification of viral transcripts in experimentally challenged birds with La Sota strain revealed that the abundance of mRNAs was significantly higher in Trachea epithelial cells than those in Harderian gland of the birds. These studies suggest that the host genome can also modulate differential gene expressions of viruses.

It is generally accepted that the F protein cleavage site is a major molecular determinant of NDV virulence^21^. Despite sharing the similar F cleavage site associated with the high virulence, virulent strains of distinct genotypes trigger markedly different pathological signs and manifestations, especially in lymphoid organs^22–24^. There may be other molecular determinants apart from F protein cleavage site related to virulence and pathogenicity of NDV-GVII strains. F and HN genes of highly virulent NDV CA02 strain were a determinant of macrophage tropism^25^. Additionally, Kai et al. shown that M, F and HN genes were related to the substantial replication of virus and strong innate immune response, contributing to the severe tissue damage and pathogenic changes^26^. Despite of significant differences in viral mRNAs transcription in different viruses, understanding of viral gene expression profiling of paramyxoviruses will help us to understand the disease production mechanisms in NDV-GVII in chickens. Few publications exist in virus-host interaction mechanisms, but they mainly focussed on very specific pathways using Northern blot, qRT-PCR and HTS in Sendai virus, Hendra virus or avirulent NDV^8,13,19,27^. This study utilised RNA-seq and qRT-PCR to identify the role of other viral genes that may contribute to pathogenicity by measuring viral mRNA genes in the infected tissue. NDV-GVII pathogenesis differs to that of other NDV genotypes, it is no information available on the viral gene expression of this highly pathogenic virus. This is the first study on viral gene expression on an experimental infection in live chicken.

## Results

After infection with virulent NDV-GVII Mega strain, spleen tissues were harvested, the RNA extracted, and reverse transcribed to cDNA. Six pairs of primers for NP, P, M, F, HN, and L were designed to amplify NDV gene fragments specifically. Resulting PCR products of the expected size were cloned into TOPO TA pCR 2.1vector and transformed into the chemically competent DH5 alpha strain of E. *coli *Cells (ThermoFisher Scientifics, VIC, Australia). Plasmids were then isolated, linearized and used to generate standard curves in absolute quantitative PCR methods. mRNA abundances (copy number) were converted from Ct values using created standard curves for each virus.

### Primer optimising and specificity of the PCR

NP, P, M, F, HN, and L specific primers were used to amplify PCR products of 112 bp, 123 bp, 112 bp, 91 bp, 86 bp and 128 bp, respectively. The annealing temperature for each primer pairs is shown in Table 1.

**Table 1:** Primer pairs and PCR efficiencies used for RT-PCR and qRT-PCR in this study.

Gene symbol	Primer sequence	Fragment size (bp)	Ta °C	Efficiency
NP	Forward: ATGAGAGCAGTGGCGAACAG	112	60	1.94
	Reverse: CCCAGTCAGTGTCGTTGTCT			
P	Forward: CATCCTTAAGTGATCTCCGA	123	54	1.99
	Reverse: CCGGTTGTGAGAGTTTATTG			
M	Forward: CTGCATATCGGGCTTATGTCCACT	112	62	1.93
	Reverse: GCACATCACTGAGCCCAACAGATA			
F	Forward: AAGCTCTCTTGATGGCAGGC	91	58	1.99
	Reverse: CCCTGTTTGAGACGAGGTGT			
HN	Forward: GGACATCTGCAACAGGGAGG	86	61	1.93
	Reverse: CACACTGCAGGACTTCCGAT			
L	Forward: GCATCCACTGTAGCACGACTATGT	128	62	1.98
	Reverse: GGTTGCGAGCTGTGGGTAATAGAA			

Melting curves for each PCR reaction were generated to test the specificity of the qRT-PCR reactions. Single melting curve peaks indicated that only target sequences were amplified (Supplementary Fig. S1).

### Standard curves and qPCR amplification efficiency

Colony PCR run was performed to screen transformed bacteria with cDNA as a positive control and water as a negative control. The presence of the insert was confirmed by Sanger sequencing using M13 and NDV specific gene primers. Standard curves were generated by SYBR Green PCR for each primer pairs using tenfold dilutions of purified and linearised plasmids in triplicate reactions, with the PCR amplification efficiencies between 93 and 99% and correlation coefficients greater than 0.993 (Table 1 and Supplementary Fig. S2).

### NDV mRNA abundances by RNA-seq and qPCR analysis

NDV-GVII strains are more lymphotropic than other strains, so the level of NDV expression in spleens was analysed. To determine the relative abundance of individual viral transcripts, RNA-seq of spleen tissue was carried out and gene expression quantified using FPKM (Fragments per Kilobase of transcript Per million Mapped reads) as the measure. FPKM adjusts counts for gene length so expression can be compared between genes.

**Figure 1:**
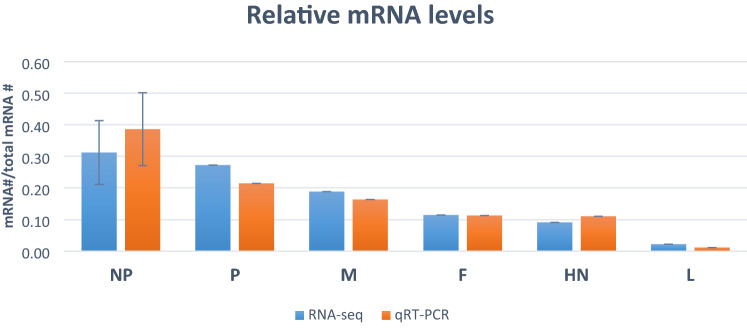
The relative mRNA abundance of viral genes measured by RNA-seq and qRT-PCR. Each bar describes individual mRNA divided by total mRNA abundance, and the error bars show the standard deviations of the means. Pairwise t-tests for each gene revealed no significant differences between RNA-seq and qRT-PCR at 5% probability.

qPCR was also used to validate RNA-seq data. Ct values for mRNA targets (NP, P, M, F, HN, L) were measured and then converted to mRNA abundance using standard curves of known concentration. Quantification of qPCR was consistent, as shown by amplification efficiencies between 93 and 99% and high correlation between replicates (R^2^ = 0.99). We used viral transcript amounts measured by RNA-seq and qRT-PCR to determine what percentage each viral mRNA contributed to the total viral mRNA (Fig. 1). RNA-seq and qRT-PCR showed a high positive correlation using linear regression (R = 0.96, *P* < 0.002), confirming the RNA-seq data's reliability. Overall, there is a progressive decline in expression of each amplicon from the 3′ to the 5′ of the genome. NP mRNAs were most abundant contributing nearly 40% of total mRNAs and gradually decreasing thereafter. Normalising to the level of NP transcript, the level of gene expression was 100:69:51:31:29:6 for NP:P:M: F:HN: L, respectively.

## Discussion

Despite having the same virulent fusion protein cleavage site and high ICPI values associated with increased virulence, the pathogenicity of the NDV strains are quite dissimilar^24,28^. This suggests that there are elements of the viral genome other than the fusion protein cleavage site that play a role in the pathogenicity of NDV-GVII. One recent study revealed that the severe pathology of NDV-GVII was in part due to the high level of virus replication and potent inflammatory response driven by the M, F and HN genes^26^. Furthermore, F and HN genes contribute to macrophages tropism of virulent NDV^25^. However, the varying role of viral mRNA expression in the virulence of NDV-GVII remains unknown. We describe the differential expression of individual viral genes of NDV-GVII in experimentally infected birds, which may be correlated with the pathogenicity of the disease and may impact on tissue response to the virus using RNA-seq and qRT-PCR.

This study the abundance of different viral mRNAs by mapping RNA-seq data to the NDV-GVII genome, separating transcripts by open reading frame and normalising counts of gene reads by viral gene length (Fig. 1). NP mRNA abundance was greater than that of L transcript, and there was slight attenuations at the NP:P and HN:L boundaries. The differential expression values of individual genes were confirmed by the absolute quantification method. Although the V gene was not specifically measured as part of gene expression, it was assumed that viral transcript counts of V gene would be expressed in a similar abundance to the P protein mRNA. Furthermore, it has been shown that the virus's transcriptional profiles vary depending on viral genotypes and host tissue. High-throughput sequencing as employed in the most recent study to investigate the paramyxovirus transcription^8^ emphasized that the transcriptional profiles between PIV2, PIV3, PIV5 and Mumps virus were substantially different, with NP highest in all except Mumps virus, PIV2 and PIV5 show a steep decline at the NP:V/P boundary, PIV3 and PIV5 at the HN:L boundary, and Mumps at the V/P:M boundary. In addition, while the NP viral transcripts for PIV2 and PIV5 are abundant, Mumps shows the same number of transcripts at NP and V/P. These suggest that NDV-GVII transcription is more similar to PIV5 than other paramyxoviruses.

The transcription of non-segmented negative, single-stranded RNA virus genomes is gradually attenuated along the genome and with the highest number of viral transcripts for gene near the 3′ end and fewest for genes near the 5′ end^4,29^. However, the viral transcript abundance for the respiratory syncytial virus (RSV) and Hendra virus (*Mononegavirales*) is not necessarily correlated with the position of a gene in the viral genome^17,30^. The transcription of the NDV genome has previously been shown to produce transcripts in molar ratios for NP:M:F:HN:L of 100:41:65:22:3^11^. While the P viral polypeptide was not detected in the previous work and why the P protein is absent was not explored, the latter *in-vivo* experiment by these authors showed that the transcriptional pattern of NDV genome followed the NP: P: F: M: HN: L order^31^. This may suggest a lack of the sensitivity and accuracy of this technique to quantify mRNA species. Another study used qRT-PCR to measure viral mRNA accumulation in cells infected with Hendra virus and found that there was a transcriptional gradient with sharp reductions in transcript abundance at the M:F and G:L boundaries ^13^. However, RNA-seq data revealed a significant attenuation in mRNA abundance at the M:F gene junction only^32^.

Estimation of NDV transcript abundance after infection with an avirulent NDV strain (La Sota) revealed that individual gene expression levels were not reduced from the 3’ to 5’ end of the genome^33^. In another recent study using qRT-PCR for gene expression quantification, illustrated that transcription of RSV (paramyxovirus) also depends on the genotype of the viruses and non-gradient patterns^17^ that are inconsistent with what is known about the expressed mRNA levels when the genome of RNA virus transcribed^14–16^.

Notably, the viral replication complex (NP, P, L) appears to be the major element of virus transcription that is associated with the gradient. Dorman et al*.* showed the significant contribution of NP, P, L in NDV virulence^34^. Moreover, in molecular evolution of *Mononegavirales*, gene expression level indicated as a major evolutionary determinant and pathogenicity for all of these viruses^18^. Several studies on the evolutionary effects of recombinant variants of Rabies virus and Vesicular Stomatitis virus revealed that the expression level of outlier proteins P and G was not a major determinant, whereas viral replication was strongly correlated with the expression level of the N and M genes^35–38^. The viral NP protein interacts with host translation machinery during NDV infection, activating multiple signalling pathways, especially PI3K/Akt/mTOR and p38MAPK/Mnk1 pathway for selective viral protein synthesis infected cells, which facilitate viral mRNA translation^39^. Moreover, Cheng et al*.* also revealed expression of NDV NP and P proteins to be important in viral replication and virulence by inhibiting of autophagy^40^. Interestingly, our recently published paper indicated that autophagy-regulated cell death was one of the most significantly inhibited pathways by NDV-GVII infection^41^, that may be associated with the highest expressed levels of NDV-GVII NP and P genes and increased virulence. The efficient replication of many viruses is influenced by the expression ratio of proteins within the viral replication complex^42^. This suggests that the potential roles of these genes and the different levels of viral gene expression could be a crucial evolutionary determinant of pathogenicity.

In conclusion, the use of high-throughput sequencing and qRT-PCR allowed us to quantitate viral mRNAs in tissues infected with virulent NDV-GVII. The different expression ratio of viral proteins may have an important role in the virulence and pathogenicity of NDV-GVII disease. While qRT-PCR is more laborious it is also convenient and easy set up in molecular laboratories, RNA-seq is more expensive and requires more bioinformatics expertise. To the best of our knowledge, this is the first study quantifying virulent NDV-GVII transcripts in vivo, revealing the differences in NDV-GVII gene expression. Further investigation is required to determine whether other genotypes of NDV have the same transcriptional pattern and whether these patterns can be associated with their pathogenicity.

## Materials and methods

### Virus preparation and collection of experimentally infected tissues

NDV isolate Chicken/Indonesia/Mega/001WJ/2013, accession number MN688613.1^43^. The challenge experiment was carried out at the biosafety level 3 (BLS3) biocontainment at Indonesian research centre for veterinary science (Bbalitvet), Bogor, Indonesia. All procedures were approved by the research and animal ethics committee of Bbalitvet institute with reference number of AH/2015/003. The animal experiments were supervised by an experienced veterinarian in accordance with the National Health and Medical Research Council (NHMRC) of Australia and the Animal Research Reporting of In Vivo Experiments (ARRIVE) guidelines 2.0. Twenty, one-day old specific pathogen-free (SPF) birds were from Caprifarmindo Laboratories (Bandung, Indonesia) and raised in isolator units at biosafety level 3 (BLS3) biocontainment at Bbalitvet. Chickens were allocated randomly into two isolators and tagged individually. A hemagglutination inhibition test was performed on the serum sample from each chicken to ensure no antibody against NDV exists in the birds. The birds were separated into two groups. The first group was a negative control and received no virus, while the second group was challenged with virulent NDV-GVII Mega strain. The birds were inoculated by intraocular and intranasal instillation with 100µL of 100 EID50 of live NDV-GVII at 35 days of age^44,45^. PBS was used as the placebo for non-infected birds. The birds were monitored daily for clinical signs, morbidity, and mortality and were then bled via a brachial vein or by cardiac puncture at the terminal step just after euthanasia. On day 3 after challenge, all surviving birds were euthanized and necropsied to collect tissue samples for RNA isolation. Spleens were collected and placed into RNA later stored at – 80 °C for later use.

### RNA extraction and cDNA synthesis

Total RNA from 50 mg of spleen from each uninfected and infected bird (10 birds for each group) was extracted using a commercial *mir*Vana™ miRNA Isolation Kit (QIAGEN, California, USA) according to the manufacturer’s instruction. These total RNAs were also used to isolate mRNA for qRT-PCR. Poly(A)^+^RNAs were selected from total RNA using ROCHE mRNA isolation kit (SigmaAldrich, Australia) according to the kit instruction.

Two-step RT-PCR was used to quantify the viral mRNA transcripts for each gene. cDNA was synthesized using the QuantiTect Reverse Transcription kit (QIAGEN *GmbH*, Hilden, Germany*)*. The optimized blend of oligo-dT and random primers enables high cDNA yields from all regions of viral RNA transcripts. Reactions carried out in 20 μl volumes in 0.2 ml thin wall, flat cap PCR tubes were including 2 steps. Firstly, a mix of 2 μl gDNA Wipeout Buffer 7 ×, 7 μl of RNA free-water and 5 μl of mRNA was prepared in tubes and incubated for 2 min at 42 °C to effectively remove any gDNA contaminations then placed immediately on ice. Next, tubes of the reverse-transcription master mix containing 1 μl of Quantiscript Reverse Transcriptase, 4 μl Quantiscript RT Buffer 5 × and 1 μl RT primer Mix was prepared on ice. Then, template RNAs from the first step were added to each tube containing reverse-transcription master mix and heated to 42 °C for 15 min and kept heating to 95 °C for 3 min to inactivate Quantiscript Reverse Transcriptase. Finally, cDNAs were placed on ice before using or stored at − 20 °C.ex

### mRNAs primer design, cloning, transformation of bacteria and plasmid isolation

Primers were designed for each viral gene (NP, P, M, F, HN, L) using the Primer-BLAST tool at NCBI. They were also aligned to the viral reference genome sequence to confirm that there were no mismatches, thus optimising accurate mRNA transcript quantification by qPCR. The PCR products ranged from 60 to 150 base pairs, ideal for maximum PCR efficiency. Fresh PCR products corresponding to each of the six viral amplicons (NP, P, M, F, HN, L) were generated by PCR and TA cloned using the TOPO TA pCR 2.1 cloning vector kit (Invitrogene, California, USA) according to the manufacturer’s instructions. The recombinant vectors were transformed into One Shot® Chemically Competent *E. coli* (Invitrogen) and 30 μl of transformed culture was spread onto LB plates containing Ampicillin (1 μl/ml). Transformants, identified as white colonies, were selected and colony PCR performed on them using M13 primers and the corresponding gene-specific primers. Colonies were cultured overnight in LB medium containing 50 μg/ml Ampicillin. Plasmid isolation was carried out using Gene Elute Plasmid Miniprep kit (Sigma-Aldrich). The presence of the insert in the recombinant clones was confirmed by sequencing using Sanger sequencing.

### qRT-PCR verification of standard templates and standard curve construction

Plasmids carrying NP, P, M, F, HN, L inserts were linearised using the *HindIII* restriction site located upstream of the insertion site^46^. Digestion products were then visualised by Gel electrophoresis to again confirm their size. DNA was quantified using a NanoDrop® ND-1000 Spectrophotometer (Thermo Fisher Scientific, Wilmington, DE). The number of plasmid molecules were determined using the following website: https://cels.uri.edu/gsc/cndna.html. In brief, number of molecules = (plasmidamount * 6.022 × 10^23^)/(plasmidlength * 1 × 10^9^ * 650). Optimal working concentrations were prepared for each plasmid; 1 × 10^10^ molecules/μl for NP; 1.7 × 10^10^ molecules/μl for P; 6.93 × 10^9^ molecules/μl for M; 8.93 × 10^9^ molecules/μl for F; 1.72 × 10^10^ molecules/μl for HN; and 7.13 × 10^9^ molecules/μl for L. Standard curves for each primer pair were generated by using at least six, tenfold dilutions of the linearized plasmid. The qRT-PCR reaction was performed in 10 µl, using 5 µl 2X SYBR Green PCR Master Mix, forward primer (1 μl, 10 μM), reverse primer (1 μl, 10 μM), RNase-free water (1 μl) and standard curves of linearised plasmid (2 μl). The thermal cycling profile included a PCR initial heat activation at 95 °C for 5 min, followed by 40 cycles of denaturation at 95 °C for 10 s, and then combined annealing/ extension at 60 °C for 30 s. To assess the specificity of amplification, a ramp of 60–99 °C was added to the melting curve step and the specificity of the reaction was confirmed by melting curve analysis. All the reactions were carried out in triplicate, including negative controls without templates. The cycle threshold (Ct) values of each dilution were measured in triplicate by The Eco real -time PCR system to generate the standard curves and were then plotted against the logarithm of the number of initial template molecules. Each standard curve was generated by a linear regression of the plotted points, creating the slope of each standard curve. PCR amplification efficiency were then calculated using the equation: E = 10^(−1/slope)^ − 1^47^. Data acquisition was carried out and analysed by EcoStudy v.50.

### qRT-PCR and NDV mRNA transcript measurements

The target sequences were amplified using the Quantitect SYBR Green PCR kit (Qiagen, Hilden, Germany) as described. The specific primers for qRT-PCR were the same as those used in generating standard curves. cDNA was diluted into RNase-free water with a ratio of 1 in 10 before using in the PCR. All negative, positive, standard curves and tested samples for the selected primer sets were dispensed to 384 well plates using an automated Corbett robot system (Corbett Research, Sydney, Australia). Reactions were performed with Applied Biosystems Real-Time PCR instruments and thermo cycle profile as described above.

The *Ct* values of the samples for mRNA targets were converted to mRNA abundances using the standard curves. As described above, each dilution of standards was measured in triplicate *Ct* values and an average *Ct* was calculated. Standards were constructed by average *Ct* values and known amounts of each linearized plasmid (molecules/rxn). For cDNAs derived from spleen samples, *Ct* values and an average of each sample were measured in triplicate and calculated. Then each average *Ct* value of samples was converted to a viral transcript abundance using the linear relationship determined for the appropriate standard curve *Ct* versus logarithm of the standard amount.

### High-throughput sequencing (HTS) and bioinformatic analyses

For each sample, 0.5 µg of total RNA was used to construct a cDNA library using an Illumina TrueSeq RNA sample preparation kit, according to the manufacturer’s recommendation (Illumina. California U.S.A.). The library sequenced on an Illumina NovaSeq S1 300 cycle instrument (Illumina. Inc, San Diego, CA, USA) to generate paired end reads of 150 bp length.

Raw RNA-seq reads were checked for quality using FASTQC v0.11.4^48^ and trimmed with TrimGalore v0.4.2^49^. Reads with a minimum length of 100 bp and where all the bases had a minimum Phred score of 10 were kept. AdapterRemoval v2.2.1 was used to eliminate adapter sequences and the reads were checked again with FASTQC^50^. The cleaned reads were mapped to a combined chicken and virus reference genome containing the NDV-GVII Mega strain sequence and chicken reference genome (Gallus_gallus.GRCg6a), using Hisat2 v2.1.0^51^. Mapped reads were then sorted with SAMtools v1.8^52^ and were counted using R package featureCounts, with genes defined using Ensembl annotation version 97^53^. Fragments per kilobase of transcript per million mapped reads (FPKM) values were used to calculate relative mRNA abundances. FPKM values normalise viral mRNA amounts produced from each individual genes to account for gene length differences, thus, the relative abundance of viral transcripts from genes of different length can be compared.

## Supplementary Information

Supplementary Information.

## Data Availability

Gene expression data were deposited in the NCBI Sequence Read Archive (SRA) under BioProject Number PRJNA675698.
