# Supplementary material for: Newcastle disease virus genotype VII gene expression in experimentally infected birds
Source: Sci Rep. 2022 Mar 28;12:5249. doi: 10.1038/s41598-022-09257-y (PMC8960812; doi:10.1038/s41598-022-09257-y)
Supplement: Supplementary file 1 — Supplementary Information. [file 41598_2022_9257_MOESM1_ESM.docx]

Fig.S1 Melting curve analysis for each PCR reaction of 6 target genes

Fig.S2 Standard curves of serially diluted linearised plasmids obtained using SYBR Green for each target sequence in order.
